# Laparoscopic management of an octogenarian adult intussusception caused by an ileal lipoma suspected preoperatively: a case report

**DOI:** 10.1186/s12957-015-0504-y

**Published:** 2015-02-22

**Authors:** Jiro Shimazaki, Takeshi Nakachi, Takanobu Tabuchi, Shuji Suzuki, Hideyuki Ubukata, Takafumi Tabuchi

**Affiliations:** Department of Gastroenterological Surgery, Ibaraki Medical Center, Tokyo Medical University, 3-20-1 Chuo Ami, Inashiki, Ibaraki 300-0395 Japan

**Keywords:** Intussusception, Lipoma, Laparoscopic surgery

## Abstract

Adult intussusception is rare and usually caused by a tumor acting as the lead point. Therefore, laparotomy should be considered for the treatment. Laparoscopic procedures for use in cases of adult intussusception have been recently reported; however, there is no consensus regarding the safety and efficacy. Here, we describe a successful case of laparoscopic management of an octogenarian adult intussusception caused by an ileal lipoma, which was preoperatively suspected. An 87-year-old male presented with progressive abdominal distention and vomiting. Contrast radiography of the small intestine showed an ileal tumor, and magnetic resonance imaging indicated a target-like mass, consistent with an ileal intussusception. The patient was suspected with an intussusception due to an ileal lipoma, and laparoscopic surgery was performed. An approximately 10-cm-long ileal intussusception with a preceding tumor was present, and partial resection of the ileum, including the tumor, was performed. Macroscopic examination of the excised specimen showed a pedunculated tumor measuring 4.0 × 3.5 × 1.9 cm with an uneven surface, yielding a histological diagnosis of lipoma. The patient had an uneventful recovery and was discharged on postoperative day 8. This successful case showed that laparoscopic surgery can be a useful, safe, and efficacious procedure for adult intussusception, even in octogenarians.

## Background

Adult intussusceptions constitute approximately 5% to 10% of all intussusceptions and are usually caused by a tumor that acts as the lead point [1,2]. While laparoscopic devices and surgical techniques have recently been improved and applied to adult intussusceptions, there is no consensus regarding safety and efficacy. This report presents a case of an octogenarian adult intussusception caused by an ileal lipoma, which was preoperatively suspected and successfully managed by laparoscopic surgery.

## Case presentation

**Figure 1:**
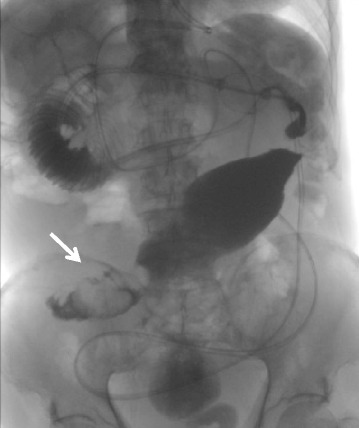
**A Gastrografin contrast radiography film revealing a filling defect.** Approximately 40 mm in diameter, in the ileum (arrow).

**Figure 2:**
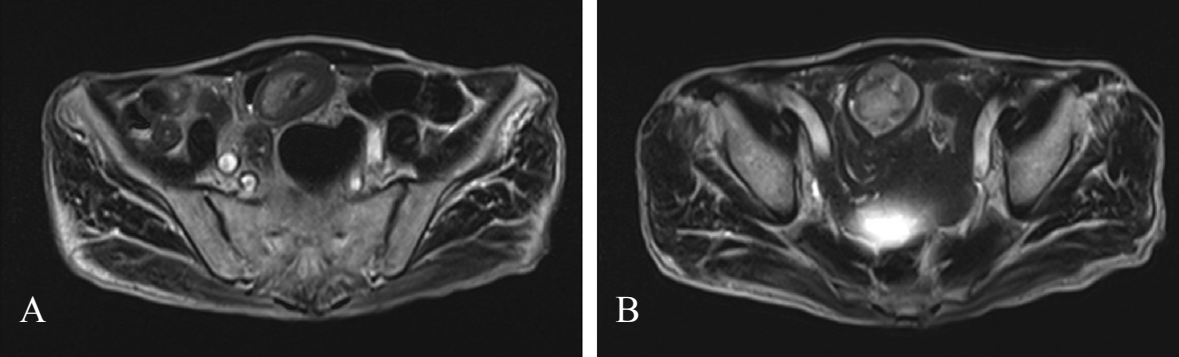
**T2-weighted magnetic resonance image.** Reveals an intussusception as a target-like mass **(A)** and a hyper-intense tumor as the lead point **(B)**.

An 87-year-old male presented at our department with progressive abdominal distention and vomiting for 2 weeks. His medical history included hypertension and revealed that he had undergone laparoscopic-assisted ileocecal resection for cecal cancer in January 2004. Physical examination revealed a distended abdomen and a tender right lower quadrant. Laboratory test results, including serum levels of carcinoembryonic antigen and carbohydrate antigen 19–9, were within normal limits. Abdominal radiography revealed a prominently dilated small intestine with some air-fluid interfaces. A long tube was inserted to reduce the internal pressure of the small intestine, and Gastrografin contrast radiography was performed. It showed an approximately 40-mm-diameter ileal tumor (Figure 1). The ileus temporally improved, and subsequent magnetic resonance imaging (MRI) showed a target-like mass consistent with an ileal intussusception (Figure 2A,B). The patient was diagnosed with intermittent intussusception with an ileal tumor, which was suspected to be a lipoma. Laparoscopic surgery was performed in May 2014.

**Figure 3:**
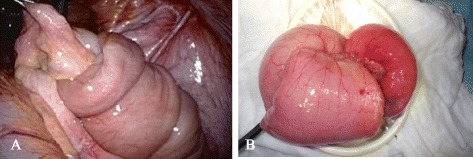
**An approximately 10-cm-long ileal intussusception with a preceding tumor. (A)** Laparoscopic image. **(B)** Intussuscepted ileum delivered from the small laparotomy.

**Figure 4:**
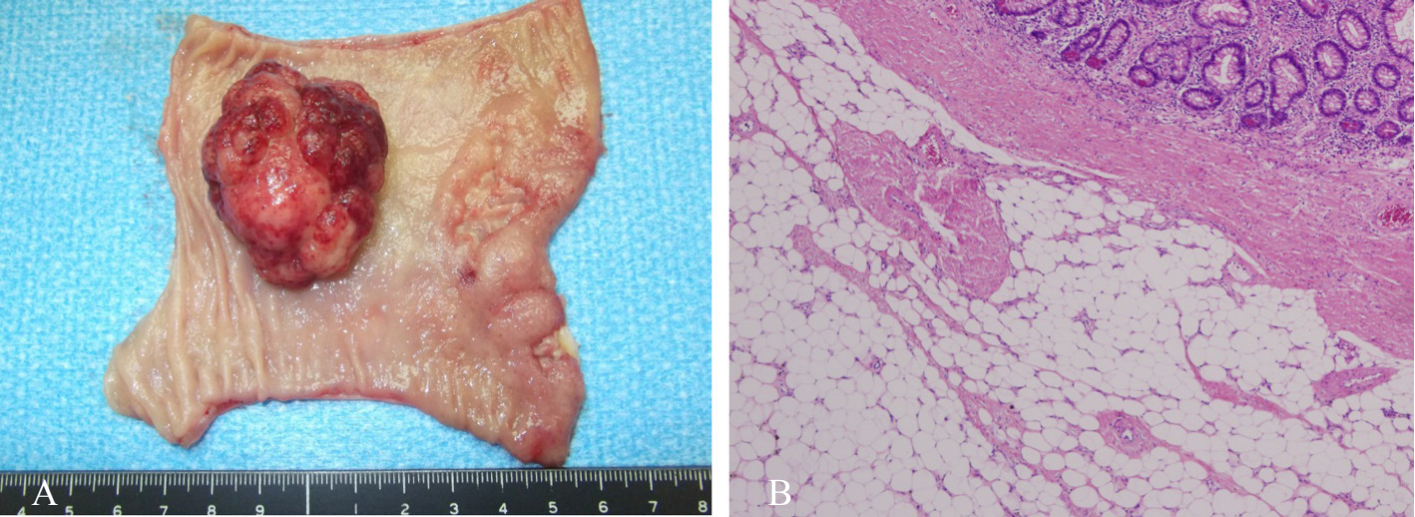
**Macroscopic examination and histological analysis of the excised specimen. (A)** Pedunculated tumor, measuring 4.0 × 3.5 × 1.9 cm, with an uneven surface and mucosal erythema. **(B)** Mature fat cells in the submucosa to muscularis propria of the ileum (hematoxylin and eosin, ×100).

The patient was placed under general anesthesia in the supine position. A 12-mm cannula for a camera was inserted in the periumbilical region by the open technique, and 10 mmHg of abdominal pressure was established by carbon dioxide insufflation of the pneumoperitoneum. Using direct laparoscopic visualization, two 5-mm ports were created in the left upper and lower quadrant for the surgeon, and two 5-mm ports were created in the right middle quadrant and just above the pubic bone for the assistant. An approximately 10-cm-long ileal intussusception with a preceding tumor was identified 40 cm proximal to the neo-terminal ileum (Figure 3A). Manual repositioning under laparoscopy did not reduce intussusception. There were sparse adhesions between the intussuscepted ileum and the retroperitoneum, which were exfoliated with a harmonic scalpel. Once the entire intussuscepted ileum had been mobilized within the abdominal cavity, a 4-cm incision was made in the periumbilicus, through which the intussuscepted ileum was delivered (Figure 3B). Partial resection of the delivered ileum including the tumor and a functional end-to-end anastomosis were performed. There were no signs of lymphadenopathy, and the liver had an unremarkable surface. Surgical blood loss was 5 ml, and operating time was 115 min. The excised tumor measured 4.0 × 3.5 × 1.9 cm and had an uneven surface with mucosal erythema (Figure 4A). Ulcerative mucosal change caused by the intussusception was observed in the oral side of the ileum from the tumor. Sectioning of the tumor revealed a solid, yellowish tissue. Histological examination of the excised tumor showed mature fat cells in the submucosa to the muscularis propria of the ileum (Figure 4B). Thickened mucosa on the tumor showed abundant micro vessels and epithelial ductal hyperplasia; however, no tumor was observed in that location.

The patient had an uneventful recovery; he passed flatus on postoperative day (POD) 1, commenced oral fluids on POD 2, and was discharged on POD 8.

## Discussion

Intussusception is rare in adults compared with that in children, and the frequency is approximately 5% to 10% of all intussusceptions [1,2]. Of the cases of intussusception in children, 95% are idiopathic with no structural lead points. On the other hand, adult intussusception is caused by an identifiable etiology in approximately 80% of the cases [3]. Previous reports have suggested that approximately 70% of the cases of adult small intestinal intussusception are caused by benign entities, such as lipomas, hamartomatous polyps, inflammatory polyps, hyperplastic polyps, and Meckel’s diverticulum [4-6]. Preoperative diagnosis is difficult because of the variability of the clinical presentation. Namikawa *et al.* [7] reviewed 50 cases of lipoma with intussusception and reported that the accuracy of preoperative diagnosis was 39.1% to 44.4%. There are several reasons why the accuracy of preoperative diagnosis is low. Patients develop intussusception as an acute abdominal condition, and there is a limit to preoperative diagnostics, particularly in the case of small intestinal intussusception. In the present case, contrast radiography showed a tumor in the ileum, and MRI findings were typical of an intussusception, that is, a target-like mass [8]. We had two reasons to preoperatively suspect that this ileal tumor was a lipoma. First, MRI showed a hyper-intense tumor in the T2-weighted image, and second, lipoma is the most common benign tumor that causes adult intussusception confined to the small intestine [9].

First treatment for adult intussusception is debatable. Many cases of adult intussusceptions are caused by an identifiable etiology; laparotomy should be considered as the first line of treatment. As a result of the marked improvement in laparoscopic devices and surgical techniques, there are reports of its use in cases of adult intussusception [10,11]. However, there is no consensus on whether laparoscopic surgery can be applied to adult intussusception with respect to safety and efficacy. Siow *et al.* [12] and Tartaglia *et al.* [13] reported that laparoscopic surgery for adult intussusception was a safe and feasible treatment, particularly in cases with an uncertain preoperative diagnosis. In the present case, laparoscopic surgery was selected for the following reasons: i) the patient’s clinical condition was stable, so he was able to undergo relaparotomy; ii) the patient was an octogenarian; therefore, we had to reduce the risk of postoperative complications, such as obstructive pneumonitis or disuse syndrome, as much as possible, which could develop from wound pain; and iii) the patient had previously undergone ileocecal resection for cecal cancer, and laparoscopic surgery would be able to exfoliate any adhesions identified in the abdominal cavity. In the English literature from the PubMed database (http://www.ncbi.nlm.nih.gov/pubmed/), there have been 14 reported cases described by the words ‘adult intussusception,’ ‘lipoma,’ and ‘laparoscopy’ including the present case (Table 1) [14-26]. The average age of the patients in these reports was 59.3 years (range 36 to 87 years), and the present case involved the oldest patient who was successfully managed by laparoscopic surgery. In most of these patients, the tumors were preoperatively diagnosed as lipomas (78.6%), and this is different from those reported by Namikawa *et al.* [7]. This difference may be attributed to the fact that laparoscopic surgery tends to be selected because of a correct preoperative diagnosis of lipoma. Eight cases (57.1%) were classified as enteric intussusceptions, which suggest that laparoscopic surgery was an effective method for adult enteric intussusception. In most cases, the number of ports was three to five; however, Chen *et al.* [25] reported on a single-port laparoscopic surgery. Although there were no data in two patients, the average postoperative stay was 7.4 days (range 3 to 15), and there were no postoperative complications. From these results, laparoscopic surgery may safely and efficaciously be used as the therapeutic method for adult intussusceptions with lipomas, even for oldest patients.

**Table 1:** **Summary of adult intussusceptions with lipoma managed by laparoscopic surgery**

**First Author**	**Reported year**	**Age (year)**	**Sex**	**Preoperative diagnosis**	**Tumor location**	**Intussusception classification**	**Tumor size (mm)**	**Number of the ports**	**Surgical treatment**	**Surgery time (m)**	**Surgical bleeding (ml)**	**Postoperative complication**	**Postoperative stay (days)**
Park KT	2001	39	F	Lipoma	Ileum	Enteric	40	3	PR	162	50	(−)	4
Ladurner R	2003	75	M	Giant intraluminal polyp	Descending colon	Colocolic	≥50	N.D.	LHC	N.D.	N.D.	(−)	10
Jelenc F	2005	56	M	Intraluminal mass	Ascending colon	Colocolic	60 × 45 × 40	N.D.	RHC	N.D.	N.D.	(−)	N.D.
McKay R	2006	63	M	Lipoma	Cecum	Ileocolic	N.D.	N.D.	RHC	N.D.	N.D.	(−)	5
Tsushimi T	2007	63	F	Lipoma	Ileum	Enteric	25 × 22 × 20	3	PR	93	N.D.	(−)	15
Lin MW	2007	47	F	N.D.	Ileum	Enteric	30 × 30	3	PR	N.D.	N.D.	(−)	4
Oyen TL	2007	54	M	Lipoma	Ileum	Enteric	N.D.	N.D.	PR	N.D.	N.D.	(−)	7
Ako E	2010	43	F	Lipoma	Ileum	Enteric	24 × 20 × 20	3	PR	78	Minimal	(−)	9
Lucas LC	2010	73	M	Lipoma	Jejunum	Enteric	21	3	PR	N.D.	N.D.	(−)	3
Ferrara F	2012	78	F	Lipoma	Jejunum	Enteric	30 × 30 × 25	3	PR	N.D.	N.D.	N.D.	N.D.
Hou YC	2012	64	F	Lipoma	Ileum	Ileocolic	N.D.	4	ICR	123	N.D.	(−)	7
Chen JH	2013	36	M	Cecal submucosal tumor or ileal lipoma	Ileocecal valve	Ileocolic	25 × 25 × 22	Single port	ICR	180	30	(−)	7
Son DN	2013	52	F	Lipoma	Cecum	Ileocolic	50 × 40	5	RHC	N.D.	N.D.	(−)	10
Present case		87	M	Lipoma	Ileum	Enteric	40 × 35 × 19	5	PR	115	5	(−)	8

F, female; ICR, ileocecal resection; LHC, left hemicolectomy; M, male; N.D., no data; PR, partial resection; RHC, right hemicolectomy.

## Conclusions

Laparoscopic surgery in experienced hands may be considered for the treatment for adult intussusception, even in octogenarians, as it is a safe and efficacious procedure.

## Consent

Written informed consent was obtained from the patient for publication of this case report and any accompanying images. A copy of the written consent is available for review by the Editor-in-Chief of this journal.

## Abbreviations

MRI: Magnetic resonance imaging
POD: Postoperative day
